# Annual nitrification dynamics in a seasonally ice-covered lake

**DOI:** 10.1371/journal.pone.0213748

**Published:** 2019-03-20

**Authors:** Stéphanie Massé, Morgan Botrel, David A. Walsh, Roxane Maranger

**Affiliations:** 1 Groupe de recherche interuniversitaire en limnologie, Département de sciences biologiques, Université de Montréal, Montréal, Québec, Canada; 2 Groupe de recherche interuniversitaire en limnologie, Department of Biology, Concordia University, Montréal, Québec, Canada; Nitte University, INDIA

## Abstract

We investigated the variability in ammonia oxidation (AO) rates and the presence of ammonia-oxidizing archaea and bacteria (AOB and AOA) over an annual cycle in the water column of a small, seasonnally ice covered, temperate shield lake. AO, the first step of nitrification, was measured *in situ* using ^15^N-labelled ammonium (NH_4_^+^) at 1% and 10% of photosynthetic active radiation during day and at the same depths during night. AO was active across seasons and light levels, ranging from undetectable to 333 nmol L^-1^ d^-1^ with peak activity in winter under ice cover. NH_4_^+^ concentration was the single most important positive predictor of AO rates. High NH_4_^+^ concentrations and reduced chlorophyll *a* concentrations under ice, which favoured AO, were coherent with high nitrate concentrations and super saturation in nitrous oxide. When targeting the ammonia monooxygenase (*amo*A) gene in samples from the photic zone, we found AOA to be omnipresent throughout the year while AOB were observed predominantly during winter. Our results demonstrate that AO is an ongoing process in sunlit surface waters of temperate lakes and at all seasons with pronounced nitrification activity observed during winter under ice. The combination of high NH_4_^+^ concentrations due to fall overturn, reduced light availability that limited phytoplankton competition, and the presence of AOB together with AOA apparently favoured these elevated rates under ice. We suggest that lake ice could be a control point for nitrification in oligotrophic temperate shield lakes, characterized as a moment and place that exerts disproportionate influence on the biogeochemical behaviour of ecosystems.

## Introduction

**Fig 1:**
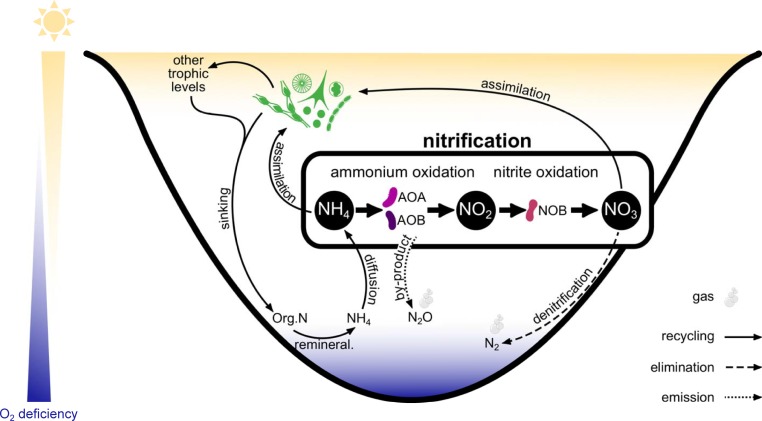
Conceptual diagram of the role of nitrification on the nitrogen (N) cycle in lakes. Optimal conditions for nitrification are represented, including low light, the presence of oxygen and high ammonium (NH_4_^+^) concentrations. By controlling the availability of N forms (mainly NH_4_^+^ and nitrate–NO_3_^-^), nitrification influences phytoplankton assimilation and community structure, as well as facilitating permanent N removal through coupled nitrification-denitrification. Ammonia oxidation (AO) can be conducted by both ammonia-oxiding archaea (AOA) and ammonia-oxidizing bacteria (AOB), whereas nitrite oxidation is conducted by nitrite-oxidizing bacteria (NOB). AO also produces nitrous oxide (N_2_O) as a by-product, a potent greenhouse gas.

Nitrification is a two-step microbial process that plays a pivotal role in the nitrogen (N) cycle, yet our understanding of the relative importance of nitrification in aquatic systems is currently heavily biased to marine systems as compared to lakes [1]. Ammonia oxidation (AO), typically considered the rate-limiting transformation [2] and the most frequently measured proxy of nitrification, first converts ammonium (NH_4_^+^) to nitrite (NO_2_^-^) and is performed by ammonia-oxidizing bacteria (AOB) or ammonia-oxidizing archaea (AOA). AO is also a chemoautotrophic process that uses dissolved oxygen as the electron acceptor. In step two of nitrification, the resulting NO_2_^-^ is converted to nitrate (NO_3_^-^) by nitrite-oxidizing bacteria. Thus, nitrification controls the relative availability of different N forms. In aquatic systems, this influences phytoplankton growth and community structure [3], but also the supply of nitrate (NO_3_^-^) for denitrification, the main N loss pathway that can mitigate eutrophication [4] (Fig 1). Furthermore, and on a global scale, nitrification has a direct effect on climate change since nitrous oxide (N_2_O) is a potent greenhouse gas and is a by-product of the AO reaction [5, 6].

Conditions traditionally considered to favor high AO rates include high NH_4_^+^ availability, low light conditions, and the presence of oxygen [1]. However, recent work has challenged some of these ideas in part with the discovery of the ability of some archaea to oxidize NH_4_^+^ to NO_2_^-^ [7] at concentrations far below the substrate threshold of AOB [8]. This explains why nitrification has been measured in very oligotrophic waters (e.g., [9–11]) and also suggests possible niche separation of AOA and AOB in space and time [8]. Methodological advances in mass spectroscopy and the use of ^15^N stable isotope tracers has also allowed direct measurement of AO rates at very low substrate concentrations [12, 13]. Hence AO has been observed in NH_4_^+^ poor waters, even within the marine photic zone (e.g. [14]). Although light inhibition of nitrification has been suggested in the past, reduced rates in sunlit surface waters relative to depth may be more a function of increased competition with phytoplankton for NH_4_^+^ [15] rather than a negative influence of light on ammonia-oxidizing organisms (AOO). Together, these findings support the more ubiquitous nature of this critical process in aquatic environments.

Despite substantial progress in our modern understanding of nitrification in oceans, our understanding in lakes is fragmented and incomplete. The presence of AOA has been observed in lakes (e.g. [10, 16, 17]) and AO rates, measured using modern techniques, have been estimated in a saline lake [18], a large great lake [10], high mountain lakes [19] and a large subtropical lake [20]. Yet, these systems are not representative of the most common naturally occurring lakes, which are small in size, located in high latitudes and seasonally ice-covered [21, 22]. Thus to fully understand the importance of nitrification in freshwaters, we must consider the rates and the players in more representative systems.

Due to seasonal changes in light flux, water column stability, and ice cover in northern lakes from temperate and boreal regions, the concentrations and availability of different N forms are very dynamic throughout their annual cycle. As such, these ecosystems represent an excellent opportunity to simultaneously follow changes in AO rates and AO community dynamics. There is increasing evidence that nitrification may be a critical process in lakes during winter under ice [23, 24]. The formation of lake ice may act as a moment and place that exerts a disproportionate influence on nitrification in lakes, i.e. acting as a control point (sensu [25]). Indeed, NO_3_^-^ and N_2_O accumulation as well as oxygen depletion has been observed under ice [26–28], suggesting nitrification is occurring. Primary producers also tend to be less abundant due to light limitation in winter under ice than during summer [29]. The clearest evidence using modern techniques that nitrification is occurring under ice in lakes, and even during the summer, comes from natural abundance stable isotopes [24]. However, direct rate measurements over the annual cycle of an ice-covered lake do not exist despite evidence that the ice-cover period is rapidly becoming shorter as a function of climate change [30, 31]. Therefore, there is an urgent need to measure and understand the role of nitrificiation in this type of lake, particularly under lake ice.

The purpose of this study was to measure the seasonal changes in the rates of AO and to characterize the presence of AOO in the surface layer of a small, seasonally ice-covered, temperate, oligotrophic lake. We hypothesized that high rates would occur in winter under ice given the ideal conditions of fall overturn entraining nutrients from hypolimnetic waters to the surface and reduced competition with phytoplankton for substrates. We also anticipated that AO should occur in the euphotic zone throughout the year as observed in marine systems (e.g.,[14, 32]) and that both AOA and AOB would be present when rates were highest.

## Materials and methods

### Site description and sampling

Sampling was carried out in Lake Croche, a pristine headwater lake at the Station de biologie des Laurentides (Université de Montréal), located on the Canadian Shield, Quebec, Canada (45°59’34”N, 74°00’34W”). Lake Croche is a small (0.179 km^2^), oligotrophic, shallow (<12 m), dimictic lake, and due to its morphometry, is subjected to hypolimnetic seasonal anoxia. The catchment area is small (1.071 km^2^) and dominated by mixed forest (>95%; [33]). Initially, monthly sampling was carried out between October 2011 and 2012 to assess interannuel variation in AO rate and AOO (herein referred to as the nitrification project, Table 1). During this project, seasonal ice-cover was present from December 10, 2011 (100% of the surface) until April 13, 2012 (0% of the surface), but thin ice prevented lake access in December, January and April for safety reasons. To complement this dataset with information on phytoplankton we also used data from a second project, where monthly or biweekly (during summer) sampling was conducted between January 2013 and September 2015. These data were part of an integrated time-series program called the sentinel lake project conducted by the Groupe de recherche interuniversitaire en limnologie (GRIL). Table 1 summarizes the two datasets used for this study. Both projects had similar sampling strategies where all N species where measured. The nitrification project did not measure chlorophyll a (chl *a*) dynamics, whereas chl *a* was measured for over two years in the sentinel project. The later study did not measure nitritication. Therefore, the sentinel project was used to fill in gaps in the nitrification project, and the variability in the concentration of the different N species bridged both studies.

**Table 1:** Summary of sampling dates, times, depths, and variables used in this study as part of two separate projects with similar datasets.

	Nitrification project	Lake sentinel project
Time span	October 2011 to October 2012	January 2013 to September 2015
Frequency	Monthly	Monthly and biweekly in summer
Depth of surface sample	10% of PAR (~3 m)	epilimnion during stratified period (0.5 m) integrated sample during mixing (0–8 m)
Depth of bottom sample	1% of PAR (~6 m)	metalimnion (3-8m)
O_2_ profile	✓	✓
PAR profile	✓	✓
NH_4_^+^	✓	✓
NO_3_^-^ + NO_2_^-^	✓	✓
N_2_O	✓ (2012 only)	✓
AO rates	✓	
Night sampling	✓	
*amo*A presence	✓	
Chl *a*		✓

Check marks indicate which measurements were made within each project. O_2_ corresponds to dissolved oxygen; PAR, photosynthetic active radiation; NH_4_^+^, ammonium; NO_3_^+^, nitrate; NO_2_^-^, nitrite; N_2_O, nitrous oxide; Chl *a*, chlorophyll *a*; AO, ammonia oxidation; *amo*A, ammonia monooxygenase subunit A gene.

For the nitrification project, sampling was conducted at the deepest part of the lake, during both day and night. Samples were collected at the depths of 10% and 1% of incoming photosynthetically active radiation (PAR) determined during the day, and the same depths were sampled at night. During the ice-free period, sampling was conducted once during the day with full sunlight and once at night, a minimum of two hours after full sunset in complete darkness. During the ice-covered period, samples were collected only during the day as very limited to no light could penetrate the ice. Vertical depth profiles of PAR and surface irradiance were measured using a LI-COR underwater LI-192 sensor and a surface LI-190 sensor during the day. During both day and night, depth profiles of temperature and dissolved oxygen were obtained using a YSI 556 multi-probe system (YSI Inc.). Discrete water samples for water chemistry, AO rates, N_2_O concentrations, and DNA analysis were collected using a plastic submersible pump (Waterra) from depths corresponding to 10% and 1% of surface PAR (I_0_). During our annual time series, 10% and 1% I_0_ corresponded on average to 3 ± 0.4 m and 6 ± 0.3 m, respectively. For night measurements, samples were collected and incubated at the same depths as during the day. During the ice-covered period, samples were collected at 3 and 6 m. For simplicity and given the month to month variability in the precise sampling depth, the two different depths will be referred to top (10% light during the day) and bottom (1% light during the day) on figures. The lake sentinel project was conducted at the same location as the nitrification project and at two depths, but sampling depths were determined based on the temperature profile. During the ice-free season, top samples represented near-surface water (0.5 m) except during the mixing period (November) where integrated epilimnetic water (0–8 m) was collected, whereas bottom samples were collected in the metalimnion (3–8 m). During the ice-covered period, water was collected underneath ice (0.5 m—top) and in hypolimnetic waters (~5 m—bottom).

### Nutrients and chl *a* measurements

For both projects, nutrients were collected and measured using similar methods. Water for NO_3_^-^ + NO_2_^-^ and NH_4_^+^ measurements were filtered immediately with 0.45 μm Acrodisc syringe filters and kept in acid washed HDPE bottles at -20°C until analysis. Nutrients analyses were conducted at the GRIL laboratory, Université de Montréal. NO_3_^-^ + NO_2_^-^ were measured spectrophotometrically using a Lachat Quickchem 800 using the Griess reaction and previous cadmium reduction (EPA 353.2). NH_4_^+^ was also measured spectrophotometrically but using the indophenol method where NH_4_^+^ reacts with hypochlorite and phenol to produce a blue compound (EPA 350.1). Samples were analyzed in duplicate and precision for NO_3_^-^ + NO_2_^-^ and NH_4_^+^ was 0.06 μmol L^-1^ and 0.2 μmol L^-1^ respectively.

During the lake sentinel project, water for chl *a* was collected in opaque HDPE bottles and was filtered in the dark onto 47mm GF/F glass fibre filters (Whatman) using a vacuum pump at gentle pressure (<7 inHg) and filters were stored at -20°C. Samples were extracted in 95% ethanol and absorbances were measured at 665 nm and 750 nm before and after acidification with 1M hydrochloric acid. Chl *a* was subsequently estimated using Lorenzen equation [34]. Samples were analyzed in triplicates and mean precision was 0.3 μg L^-1^.

### N_2_O concentrations

The partial pressure of N_2_O (*p*N_2_O) was measured from February to October 2012 during the nitrification project, and for the complete time span of the lake sentinel project using the same technique. *p*N_2_O measurements were obtained using headspace equilibration [35] where a 1.12 L glass bottle was filled using the overflow teachnique with lake water from both depths, hermetically sealed and 0.12 L of water was removed from the sealed bottle and replaced with ambient air. The bottle was mixed vigorously for 2 min to achieve headspace equilibration with water. Nine millilitres of air were then sampled in triplicate using an airtight syringe and transferred into 9 mL pre-evacuated glass vials capped with an airtight butyl seal. Ambient air samples were also collected. Samples were analyzed using a Shimadzu GC-2040 gas chromatograph, with a Poropaq Q column to separate gases. N_2_O concentrations were determined using an ECD detector. Concentration in the water (*C*_*water*_) and the expected saturation concentration in water at air equilibrium (*C*_*eq*_) were corrected for before and after equilibrium sample temperature and ambient atmospheric pressure. N_2_O deviation from saturation (Δ N_2_O) was calculated as *C*_*water*_*−C*_*eq*_. During the winter, we assumed no exchange with the atmosphere and used the *C*_eq_ of the first winter sample for the Δ N_2_O calculations.

### In situ AO rates using ^15^N-NH_4_^+^

As part of the nitrification project, AO rates were quantified using ^15^N tracer experiments from October 2011 to October 2012. This method measures the isotopically enriched portion of the NO_2_^-^ pool after the addition of ^15^N-labelled NH_4_^+^. Four 10% HCl-washed and ultrapure water-rinsed clear 300 mL BOD bottles were filled with lake water from each depth using the overflowing technique. Each bottle received a tracer addition of NH_4_^+^Cl (99% ^15^N), corresponding to 5% of the ambient NH_4_^+^ concentrations based on measurements at both depths that were taken one month before each experiment. When ambient concentrations were too low to calculate tracer addition, 0.05 μmol L^-1^ of ^15^N-labelled NH_4_^+^ was added [36]. Although the ^15^N-labelled NH_4_^+^ spike was designed to be ≤ 10% of ambient lake NH_4_^+^ concentration, this was not achieved in all incubations. Tracer addition was higher than 15% of ambient lake NH_4_^+^ concentration in 9 incubations (25 to 100% of *in situ* concentration) and might have resulted in an overestimation of rates for those dates (only during the ice-free season, S1 Table). Carrier, Na^14^NO_2_^-^, was also added to increase ambient levels by 0.25 μmol L^-1^ to allows the recently produced NO_2_^-^ to be diluted into a larger unenriched pool [36]. This increase in overall NO_2_^-^ concentrations was also required for isotopic measurements, because *in situ* concentrations were too low. For each treatment, two bottles were incubated for 3–4 h directly in the lake in transparent nets at their respective sampling depths, whereas for time zero, duplicate samples were immediately filtered after tracer addition. Efforts were made during sampling to minimize light exposure in order to reduce risk of AO rates bias. Tracer and carrier solutions were purposely kept colder than sample temperature prior to addition to ensure that they sank to the bottom of the bottle and were not displaced by capping the bottle. After incubation, water samples were filtered through pre-combusted (450°C for 4 h) GF/F glass fibre filters (Whatman) under low pressure (<7 inHg). The filtrate was then kept at -20°C until isotopic analysis. Supplementary material S1 Table summarizes conditions of AO rates assays, including detailed incubation times and achieved NO_2_^-^ and NH_4_^+^ concentrations in bottle.

The δ^15^N of NO_2_^-^ was determined using the sodium azide method which converts NO_2_^-^ into N_2_O of McIlvin and Altabet [13]. Briefly, 10 mL sample aliquots were placed in pre-combusted (450°C, 4 h) glass vials and capped with airtight butyl seals. A fresh solution of 2 M sodium azide and 20% acetic acid, combined in a 1:1 ratio (v/v), was purged with helium for 30 min to remove any N_2_O. A subsample of 0.6 mL of this mixture was then added by syringe in each of sample aliquots, which were subsequently shaken vigorously and incubated at 30°C for 1 h. The reaction was neutralized by adding 0.38 mL of 10 M sodium hydroxide. The isotopic analyses of N_2_O were performed at the GEOTOP, Geochemistry and *Geodynamics Research Center*. Specifically, the N_2_O was purged from sample aliquots with helium, trapped and concentred with liquid nitrogen, and purified using a Micromass TraceGas inlet. The isotopic composition of N was measured using a Micromass Isoprime continuous flow isotope ratio mass spectrometer. Replicate measurements of internal reference materials yielded uncertainties better than 0.4‰. Samples were calibrated and blank corrected using four in-laboratory internal isotopic NO_2_^-^ standards (δ^15^N -76.9‰, δ^15^N -35.6‰, δ^15^N 1.7‰, and δ^15^N 36.7‰). Standards were run before, at 15 sample intervals and at the end of each run. The detection limit was ~ 2 nmol N (corresponding to ~ 250 nM on the basis of the volume of sample used). Based on the analysis of 10% replicate samples, the reproducibility (1 σ) of δ^15^N was better than 6.8‰. All N isotopic data were reported relative to the Atmospheric Air Primary Reference Scale.

Rates of ^15^NH_4_^+^ oxidation were determined from the isotopic composition of NO_2_^-^ measured at the beginning and end of single end-point ^15^N tracer experiments using the following equation modified from Carini and Joye [18] and Horak et al. [37]:

$$r\;\left(nmol\;L^{‐1}\;day^{‐1}\right)=\left(2\;*\;\left[n^{15}NO_{2}{}^{‐}\left(f\right)-n^{15}NO_{2}{{}^{‐}}_{\left(i\right)}\right]\;*\;NO_{2}{{}^{‐}}_{\left(i\right)}\right)/\left(t\;*\;\alpha \right))$$ (1)

where r is the AO rate (nmol L^-1^ d^-1^), n^15^NO_2_^-^_(f)_ is the atom percent of ^15^NO_2_^-^ at the end of the incubation, n^15^NO_2_^-^_(i)_ is the initial atom percent of ^15^NO_2_^-^, NO_2_^-^_(i)_ is the initial ambient NO_2_^-^ concentration in the incubation bottle immediately after tracer and carrier additions, t is the incubation time, and α is the proportion of ^15^NH_4_^+^ from the NH_4_^+^ concentration in the incubation bottle (calculated as the concentration of ^15^NH_4_^+^ added divided by the sum of the tracer concentration plus ambient NH_4_^+^ concentration). Our hourly rates were converted to daily rates to have estimates comparable to previously published values. Because the method measures ^15^NO_2_^-^, the right end product of the equation was multiplied by two to account for each N atom of the N_2_O molecule which received one N atom from the non-enriched azide and one atom from the ^15^NO_2_^-^ pool [37]. NO_2_^-^ concentrations were measured spectrophotometrically similarly to NO_3_^-^ + NO_2_^-^, but omitting the cadmium reduction step. We observed that NO_2_^-^ concentrations decreased during some incubations, likely due to uptake by phytoplankton, or oxidation to nitrate [36]. For this reason, we used initial instead of final ambient NO_2_^-^ concentrations in rate calculations and assumed that NO_2_^-^ consumers would favour the uptake of the lighter isotope and not have an effect on δ^15^N of NO_2_^-^. However, this likely resulted in an underestimation in AO rates as any NO_2_^-^ production was not accounted for. ^15^NO_2_^-^ was depleted relative to background in some cases, resulting in negative estimates of AO, which we considered as undetectable rates. Finally, to minimize the dilution of the substrate pool, we choose reatively short incubation times (3–4 hours) [36]. We therefore considered NH_4_^+^ regeneration to be minor and did not account for it during our incubations. If rapid substrate regeneration occurred, the labeled fraction would be diluted resulting in an underestimate of the rates [38, 39].

### DNA extraction and PCR amplification of *amo*A gene

DNA extractions were conducted only on day-time samples collected during the nitrification project time series. Discrete water samples from both depths where prefiltered through a 20 μm nylon mesh to remove zooplankton and were then sequentially filtered through a 3.0 μm pore size polycarbonate membrane and 0.2 μm pore size Sterivex unit (Millipore, USA). Sterivex units and 3.0 μm pore size filters were preserved by adding 1 mL of lysis buffer solution (40 mmol L^-1^ EDTA [pH 8], 50 mmol L^-1^ Tris [pH 8.3] and 0.75 mol L^-1^ of sucrose). After flash-freeze, samples were stored in liquid nitrogen until final storage at– 80°C. DNA was extracted from 0.2 μm pore size Sterivex units using a modified protocol from Zaikova et al. [40] as described in supporting information (S1 Text). The archaeal and bacterial *amoA* genes were amplified from day-time samples at both depths. For the archaeal *amo*A gene (635 bp fragment), amplification was performed using the primer set Arch-amoAF (5’-STA ATG GTC TGG CTT AGA CG-3’) and Arch-amoAR (5’-GCG GCC ATC CAT CTG TATGT-3’)[41]. For the bacterial *amo*A gene, amplification was performed using the primer set amoA-1F* (5’-GGG GHT TYT ACT GGT GGT-3’) [42] and amoA-2R (5’-CCC CTC KGS AAA GCC TTC TTC-3’) [43]. The PCR conditions are described in supplementary materials.

### Sequencing and phylogenetic analyses

Clone libraries of archaeal and bacterial *amo*A genes were generated using PCR techniques for four and three discrete samplings respectively, from the nitrification project time series as described in S1 Text. Archaeal and bacterial *amo*A sequences were compared and aligned with published sequences obtained from the NCBI nucleotide database (GenBank). All phylogenetic analyses were conducted using *MEGA* version 5. The best-fit models of nucleotide substitution for the construction of phylogenetic trees were statistically chosen based on the Bayesian Information Criterion (BIC) using jModelTest [44, 45]. Archaeal and bacterial phylogenetic trees were inferred using maximum likelihood method based on the Hasegawa-Kishino-Yano model [46] and the Tamura-Nei model [47], respectively. Bootstrap analysis was used to estimate the confidence of each node (1 000 replicates). Representative sequences for archaeal and bacterial clones recovered from the Lake Croche water column were deposited in GenBank under the accession numbers LN997817 to LN997833.

### Statistical analysis

All computations and statistical analyses were performed using R 3.4.0 [48]. For certain tests, variables were transformed to meet normality assumptions. Differences in AO rates among seasons were assessed using the Kruskal-Wallis test and a pairwise Dunn test with holm correction for p-value was subsequently used to identify which groups were significantly different. The same procedure was applied to assess differences in ΔN_2_O among seasons. To test differences in AO rates between depths and over the diel cycle (i.e., day and night), a paired t-test was used.

To explain the variability of AO rates, least square multiple linear regression (MLR) with forward selection was performed between AO rates and the environmental variables (NH_4_^+^, dissolved oxygen, water temperature and proportion of surface radiation—I_z_/I_0_). In addition, a univariate regression tree (URT) analysis of AO rates was performed to test for possible non-linear relationships between AO rates and the set of explanatory variables which could identify thresholds in higher activity. We used the same environmental variables, but this time not transformed, with the addition of season, depth, and day and night coded as factors. The most parsimonious tree was chosen based on the lowest cross-validation error. NO_3_^-^ + NO_2_^-^ was not included in these statistical analyses since it is the final product of nitrification, but the Pearson correlation coefficient between AO rates and NO_3_^-^ + NO_2_^-^ was assessed.

## Results

### Physico-chemical characterization of the lake

During the 13-month time series that included AO measurements, Lake Croche followed a classic dimictic stratification pattern for a north temperate lake, with overturn periods in November and April, and the onset of thermal stratification occurring in May (S1 Fig). The light regime for selecting sampling depths (i.e. 10% and 1% I_0_) was fairly consistent during the open water period at 3 and 6 m depth, respectively. During winter the same depths were sampled for consistency, but no light was able to penetrate the ice. Oxygen was present at all periods (S1 Fig) and a seasonal oxycline, fluctuating between 6 and 9 m, was observed between May and September. Oxygen concentrations were particularly low at the 1% light levels in August and September but were always >2.8 mg L^-1^.

### N dynamics and factors influencing AO rates

**Fig 2:**
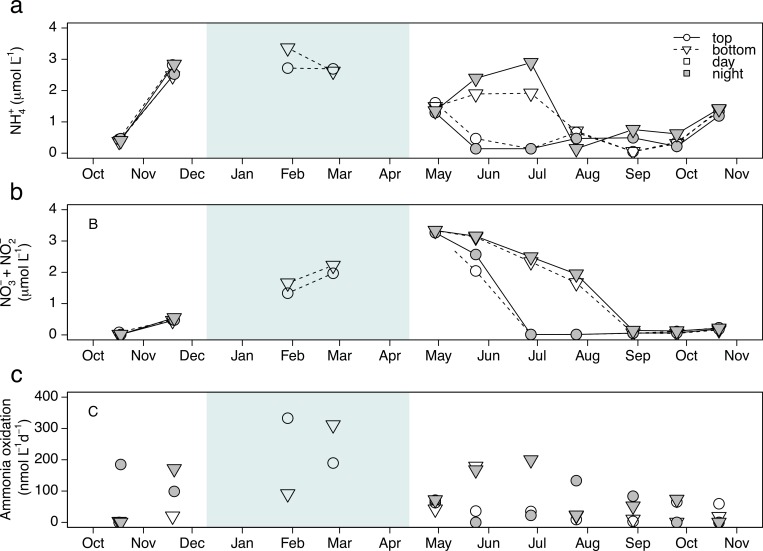
Temporal variation of ammonium concentrations (NH_4_^+^, μmol L^-1^) in (a), nitrate + nitrite concentrations (NO_3_^-^ + NO_2_^-^, μmol L^-1^) in (b), and ammonia oxidation rates (AO, nmol L^-1^day^-1^) in (c) at both depths sampled and during day and night. Top and bottom refer to the depth where 10% and 1% of the incident light was measured during the day. The period where the lake was covered by ice is depicted by a light blue rectangle.

Dissolved NH_4_^+^ concentrations ranged from undetectable to 3.4 μmol L^-1^ at both sampling depths (Fig 2A) from October 2011 to October 2012. Break down of thermal stratification in November supplied the upper layer with NH_4_^+^-rich water prior to ice-cover and elevated NH_4_^+^ concentrations for this oligotrohic system were observed throughout the winter (${\bar{x}}_{\mathrm{winter}}$ = 2.8 μmol L^-1^). After ice-out, NH_4_^+^ concentrations were slightly lower than those observed in winter but similar throughout the water column due to spring overturn. At the beginning of the stratification period (May and June), mean surface concentrations were lower than those at depth, 0.2 μmol L^-1^ versus 2.3 μmol L^-1^ respectively with the highest NH_4_^+^ concentrations measured at the deepest site (i.e., 7 m) in June (Fig 2A). From the end of July until overturn, NH_4_^+^ concentrations were consistently low at the surface and at depth (<0.8 μmol L^-1^ and average of 0.4 μmol L^-1^). Concentrations of NO_3_^-^ + NO_2_^-^ followed a different pattern to that of NH_4_^+^. Near-zero concentrations were observed in October and November (Fig 2B). NO_3_^-^ + NO_2_^-^ concentrations gradually increased throughout the winter and reached peak concentrations at both sampling depths at the end of April (3.3 ± 0.04 μmol L^-1^). Concentrations declined during the stratified period and returned to minimal values in autumn. Measured ΔN_2_O (deviation from equilibrium with air) at both depths ranged from -0.06 to 0.12 ppm. Highest ΔN_2_O was observed under the ice in February, but were not statistically different from other seasons (S2 Fig).

**Fig 3:**
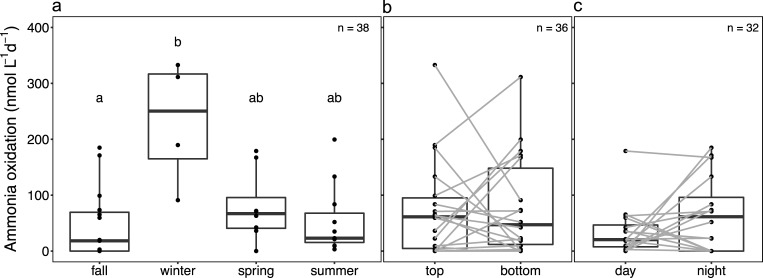
Box and dot plots of ammonia oxidation (AO) rates according to (a) the season and (b) the depths and (c) the diel sampling time. In b and c, grey lines between groups represent paired sampled. Medians are denoted by central lines and are bounded by the 25th and 75th percentiles. Whiskers show 10th and 90th percentiles. Dots outside boxes and whiskers are outliers. Different letters represent significant difference at level *p* = 0.05 among groups based on non-parametric pairwise Dunn’s test.

AO rates across seasons and depths ranged from undetectable to 333 nmol L^-1^ d^-1^, with a peak in activity observed under the ice (Fig 2C). Interestingly, AO activity was observed throughout the photic zone of the water column all year long. Across seasons, winter rates were highest overall but only statistically significantly greater than those measured in autumn (Fig 3A; *p =* 0.009). Compared to mean fall and summer rates (46.3 and 53.8 μmol L^-1^ d^-1^), mean winter rates (231.2 μmol L^-1^ d^-1^) were 4 and 5 times greater, respectively. No significant difference in AO rates was observed between depths using paired t-test (Fig 3B; *p* = 0.95). However, a pairwise comparison by date showed that AO rates were higher at bottom depth 55% of the time. AO rates measured at night tended to be higher than those measured during the day, but this pattern was also not statistically significant (Fig 3C; *p* = 0.14). Again, however, a pairwise comparison showed that AO rates were higher at night than during the day 69% of the time.

**Fig 4:**
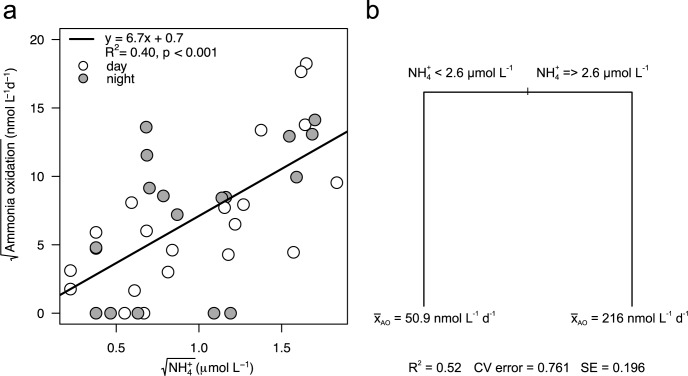
Relationships between ammonium (NH_4_^+^) concentrations and ammonia oxidation (AO) rates using (a) ordinary least square regression and (b) univariate regression tree (URT). In a) grey circles are night samples and white circles day samples. In b) the split is labeled with the variable and the concentration that determines the split. Each node is labeled with the mean rating and the number of sites in the group.

We used a MLR approach to determine which variables could best explain the variability in AO. NH_4_^+^ was the single positive predictor of AO rates (Fig 4A) and no other variables entered the model. Nevertheless NH_4_^+^ concentration alone explained 40% of the variance in measured rates (Fig 4A). NO_3_^-^ + NO_2_^-^ concentrations and temperature were also correlated to AO rates (*r*_NO3+NO2_ = 0.41, see supplement material S2 Table), albeit weakly. In the case of NO_3_^-^ + NO_2_^-^, it is the product of nitrification, so a relationship was expected. For temperature, the negative correlation was an artefact of the winter effect. To reveal any non-linear relationship and potential thresholds between AO rates and environmental data, we also used URT analysis. Again, AO rates could be predicted using NH_4_^+^, which explained 52% of the variance (Fig 4B). The two-leaf tree was shaped by NH_4_^+^ concentrations with higher rates found when NH_4_^+^ concentration was higher than 2.6 μmol L^-1^, which occurred primarily during winter.

### Seasonal dynamics of chl *a* and nutrients

**Fig 5:**
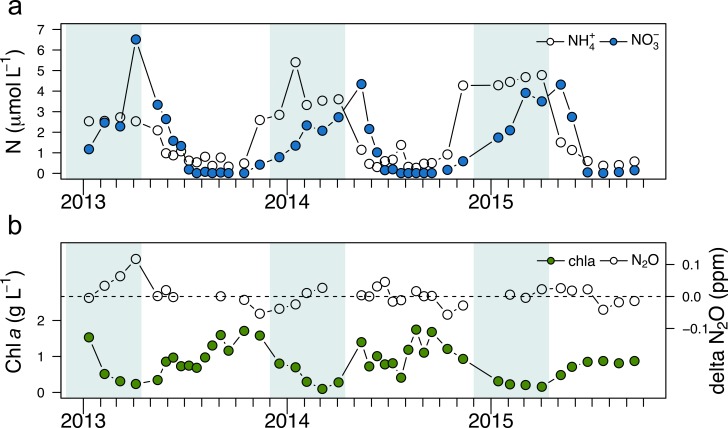
Temporal variation in surface water a) ammonium (NH_4_^+^) and nitrate (NO_3_^-^ + NO_2_^-^) and b) chlorophyll *a* and delta N_2_O between January 2013 and September 2015 from sample collected as part of lake sentinel project in lake Croche. Dotted line in panel b) represents N_2_O concentrations at equilibrium with air. The period where the lake was covered by ice is depicted by a light blue rectangle. Tick mark spacing represent month starting in January.

During the period when AO rates were measured, chl *a* concentrations were not (Table 1). However, as part of the lake sentinel project Lake Croche was visited monthly between 2013 and 2015 where nutrients, chl *a*, and N_2_O concentrations were all measured simultaneously (Fig 5A and 5B). Patterns of NH_4_^+^ and NO_3_^-^+NO_2_^-^ measured between 2013 and 2015 in surface waters emulated what was observed in 2011 and 2012 during the nitrification project. The greater detail in the resolution under the ice showed that peaks in NH_4_^+^ preceded those of NO_3_^-^+NO_2_^-^ (Fig 5A), supporting the notion that AO and nitrite oxidation were occurring under the ice. The pattern for ΔN_2_O is less clear, but typically the greatest deviations from saturation either occurred under the ice or increased during the ice-covered period (Fig 5B). By comparison, chl *a* concentrations showed an inverse pattern to nutrients (Fig 5B), with peaks in summer and negligible concentrations under the ice. Mean under-ice chl *a* concentrations (0.4 μg L^-1^) for surface samples were 10% of mean summer values (4.0 μg L^-1^). Patterns at depth were similar, although nutrient concentrations and N_2_O deviations were higher as a function of hypolimnetic supply and potential N_2_O production from denitrification (S3 Fig).

### Detection and diversity of AOO

**Fig 6:**
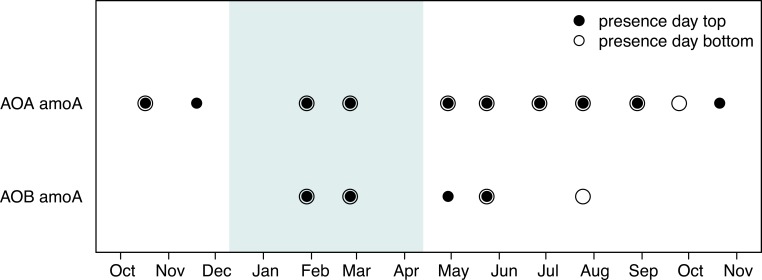
Presence of archaeal and bacterial *amo*A gene fragment at both depths during the day. Presence is assumed when PCR amplicon was obtained. The period where the lake was covered by ice is depicted by a light blue rectangle. AOA ammonia-oxidizing archaea; AOB ammonia-oxidizing bacteria.

The diversity and community structure of AOA and AOB was monitored for seasonal changes using the *amo*A gene as a marker in 2011 and 2012 when AO rates were measured directly. Day-time samples were explored for diversity on all dates. Archaeal *amo*A gene fragments were detected in approximately 85% of samples and were present throughout the year in the water column (11 months; Fig 6). In contrast, amplification of bacterial *amo*A gene was successful for less than half the dates (5 of 11 months; Fig 6). However, AOB amplification intensity was highest in samples collected under ice during January and February.

To reveal the phylogenetic composition of AOA, archaeal *amo*A amplicons were analyzed from four deep samples, one from each season. All archaeal sequences retrieved from the water column of the oligotrophic lake were members of *Nitrosotalea* (also referred to as SAGMGC-1 or group I.1a associated) and *Nitrosopumilus* (also referred to as Thaumarchaeal marine group I.1a) clusters based on the nomenclature of Pester et al. [49](S4 Fig). At higher phylogenetic resolution, most sequences fell into one of two environmentally coherent subclusters (*Nitrosotalea* subcluster 1.1 and *Nitrosopumilus* subcluster 1.1), both containing sequences recovered from other freshwater environments. The sequences from Lake Croche were closely related (> 93% identity within the three following subclusters respectively) to the three most abundant AO Thaumarchaeota ecotypes (*Nitrosotalea* 1.1a, 1.1b and *Nitrosopumilus* 1.1) recently reported in high mountain lakes [50, 51]. Finally, one of the *amo*A sequences collected in winter grouped with *Nitrosopumilus* subcluster 5.1, a cluster dominated by freshwater and ground water sequences [17].

Bacterial *amo*A sequences were generated from samples collected during the day from bottom depth from the three months that exhibited the highest *amo*A gene amplification per season (i.e. February, May and July). The bacterial *amo*A gene was not detected in autumn samples. Six sequences matched the beta-proteobacterial *amo*A gene. Based on the nomenclature of Francis et al. [52], phylogenetic analysis revealed that these six sequences fell into two clusters (*Nitrosospira*-like clusters A and B) from which no cultured representatives are known (S4 Fig). Interestingly, the first cluster contains the majority of previously reported *amo*A beta-proteobacterial sequences retrieved from two freshwater lakes [53], a few sequences from oligohaline habitats in Cheasapeake Bay [52] and from the roots of rice plants [54].

## Discussion

This study is the first to our knowledge to simultaneously quantify the *in situ* AO rates and assess the presence and the diversity of AOA and AOB across seasons in a small ice-covered temperate, oligotrophic lake. Four major outcomes were identified: (1) AO was observed at 10% and 1% of surface PAR and throughout all seasons; (2) NH_4_^+^ concentrations exerted the strongest influence on rates; (3) AOA were observed throughout the year and likely play a dominant role in nitrification in oligotrophic lakes and (4) lake ice appears to act as a control point for AO. This is likely due to the highest availability of NH_4_^+^ under ice, the presence of both AOA and AOB and reduced competition with phytoplankton for this critical substrate as a function of light limitation.

### AO ubiquity and seasonality in response to NH_4_^+^

Recent research has shown that marine AO communities are active in the photic zone, challenging the long-standing paradigm that nitrification should be photoinhibited in sunlit waters [55–57]. Indeed, peaks of both nitrifying activity (e.g. [14, 32, 58, 59]) and *amo*A expression [60] have been observed in the ocean surface. This finding challenges our interpretation of new versus regenerated production in the ocean, as significant NO_3_^-^ production through nitrification in the euphotic zone would suggest that new production would be overestimated [32]. Our study confirms that AOO were present throughout the year and that AO occurs at relatively high rates, even in the sunlit waters of a north temperate lake.

Although AO rates tend to increase with depth with maximum rates typically observed at the bottom of the euphotic zone in marine ecosystems [32, 58], light is apparently less of the limiting factor for AOO than competition for substrates with phytoplankton [15]. Our results support this notion. AO rates were not significantly different between samples collected at the 10% and 1% light levels, nor between night and day on dates sampled during the ice-free period. However, a pairwise comparison showed that in over half these cases, rates were higher either at depth or at night, when N uptake by phytoplankton would be reduced [61]. Active nitrification during the ice-free season also suggests that a fraction of the N available to surface phytoplankton communities in small lakes would come from nitrification. Indeed, between 5% and 30% of NO_3_^-^ assimilation by phytoplankton N was sustained by nitrification in another small stratified lake during summer [24]. Stratification in small lakes may be essential to support nitrification in surface waters, as NH_4_^+^ could be chronically supplied via diffusion from rich anoxic hypolimnetic waters. In the late summer, however, lower AO rates at the bottom depth were observed, which may be a function of competition with phytoplankton since Lake Croche is known to have metalimnetic peak (deep chl *a* maximum; [62]).

Interestingly, seasonal AO rates measured in our study lake almost span the range of water column rates reported in the literature (Table 2). Although higher rates have been observed in a few other systems, the under-ice AO rates in lake Croche are among the highest reported (Table 2). The wide variation in rates observed in this study is largely due to the stratification and physical mixing dynamics of this ecosystem, which results in highly variable surface water NH_4_^+^ concentrations throughout the year (0–3.36 μmol L^-1^). Ambient NH_4_^+^ concentrations alone explained 40% of the variability in AO rates in Lake Croche (Fig 4A). This tight coupling has also been observed in other systems [10, 32, 63]. In fact, AOO have been shown to respond rapidly to increase substrate concentrations by immediately enhancing their *amo*A transcriptional activity [60, 64]. AO rates in this study were also positively correlated, but to a lesser extent, with NO_2_^-^+NO_3_^-^ concentrations. This relationship was somewhat expected given that maximum rates were measured during the cold winter months when both NH_4_^+^ and NO_2_^-^+NO_3_^-^ concentrations were highest, showing a tight coupling between substrate and product under conditions where competition with phytoplankton is reduced and AOB are present.

**Table 2:** Comparison of nitrification rates reported for pelagic environments. All rates were obtained by measuring the oxidation of ^15^N-labeled NH_4_^+^.

Location	Type of system	Depth	NH_4_^+^ (μmol L^-1^)	Nitrification rate (nmol L^-1^ d^-1^)	Light condition	References
Lake Croche, Quebec	small oligotrophic lake	Bottom of the photic zone	0–3.36	0–333	*In situ*	This study
Lake Superior, US and Canada	large freshwater lake (oligotrophic)	2–150 m	0.09–1.11	0–83*	Dark	Small *et al*. (2013)
Taihu Lake, China	large and shallow freshwater lake	Surface (0.2 m) and near-bottom water (2 m)	0.26–131	2–3750*	*In situ*	Hampel et al 2018
Mono Lake, California	alkaline and saline lake	Surface to oxycline layer	0–20	4–480*	Dark	Carini and Joye (2008)
Scheldt estuary, Netherlands and Belgium	freshwater part of the estuary	2 m	≤ 150	3600–16800**	Dark	Andersson *et al*. (2006)
	marine part of the estuary		≤ 25	0–480**		
Hood Canal, Washington	fjord	0–120 m	0–0.646	0–550*	Dark	Horak *et al*. (2013)
Saanich Inlet, British columbia	fjord	Between photic zone and the oxic-anoxic interface (~140 m)	0–3.5	0–120	Dark	Ward and Kilpatrick (1990)
Gulf of California	coastal waters	Upper water column (30–60 m)	0.01–0.05	0–93*	*In situ*	Beman *et al*. (2008)
Monterey Bay, California	coastal waters	Photic zone and 3 depths below	Undetectable	20–80	*In situ*	Ward (2005)
Central California Current	open ocean	Photic zone	0–1	2–210*	Dark	Santoro *et al*. (2010)
Arabian Sea	open ocean	60–0.1% Io	≤ 1	5–100	*In situ*	McCarthy *et al*. (1999)
Arabian Sea	open ocean	40–1500 m	undetectable	0–22	Dark	Newell *et al*. (2011)

Values with * correspond to rates that were calculated based on the accumulation of the ^15^N in both NO_2_^-^ and NO_3_^-^ pools, instead of NO_2_^-^ pool. Values with ** correspond to rates that were calculated based on the accumulation of the ^15^N in the NO_3_^-^ pool only. The type of system, the depth sampled, the range of ambient ammonium (NH_4_^+^) concentrations and the light conditions during incubation are reported.

### Variation in ammonia-oxidizer community composition

The seasonal changes in NH_4_^+^ concentrations also influenced the ammonia oxidizer community composition. To assess AOO diversity specifically, we targeted the *amo*A gene as a marker rather than the 16S rRNA gene. AOA were omnipresent throughout the year, while AOB were only intermittently detected (Fig 6). Unfortunately, we were unable to quantify AOA or AOB abundances by qPCR-based approaches because PCR amplification was quite low in our study. Nevertheless, our findings agree with the growing evidence that AOA are more likely to dominate AO communities in oligotrophic aquatic ecosystems, when NH_4_^+^ concentrations are typically less than 2 μmol L^-1^ (e.g., [9, 10, 51, 59, 65]). Physiologically, it has been shown that the AOA *Nitrosopumilus maritimus* have half-saturation constants (*K*_m_) approximately 300 to 1,000 times lower than that of AOB (AOA: [8], AOB: [66, 67]), providing them with a competitive advantage under low substrate conditions. In marine systems, where AOA are often omnipresent and dominant, the K_m_ of natural communities are close to that of *Nitrosopumilus maritimus*, showing the competitive advantage of AOA over AOB in natural oligotrophic systems [37, 68]. Recent work in freshwater alpine lakes has identified new archaeal *amo*A sequences, which cluster with “*Candidatus* Nitrosotalea devanaterra” (from acid soil) in the *Nitrosotalea* group (also referred as SAGMGC-1 or group I.1a associated) [50, 51, 69]. In Lake Croche, all the archaeal sequences observed, except one found within the *Nitrosopumilus* cluster [70], are very closely related to the three most abundant ammonia-oxidizing Thaumarcheota ecotypes reported in these high mountain lakes (S4 Fig) suggesting these ecotypes may be ubiquitous in freshwaters.

### Winter and lake ice as a control point for AO

From our study, it appears that periods with peaks in ambient NH_4_^+^ concentrations allowed AOB to co-occur with AOA, at least in Lake Croche. Interestingly, co-occurrence was prominent under ice, when high dissolved inorganic N and O_2_ concentrations combined with low light intensities that reduced competition with phytoplankton creates ideal conditions favouring nitrification (Fig 2, S1 Fig). Relatively high nitrifying activity has been previously reported in the water column of a temperate lake [23], as well as in the coastal Arctic Ocean during winter under ice [71]. Observed NO_3_^-^ accumulation and dissolved O_2_ depletion in a series of lakes also suggests occurrence of high-nitrifying activity under ice [27, 28]. By using modern techniques that measure AO directly, our study shows that the process mitigating this NO_3_^-^ accumulation and O_2_ loss is indeed nitrification, but furthermore we show that AOB may be additional players to this process in the under-ice environment of oligotrophic shield lakes.

AOB affiliated with the *Nitrosospira* lineage (S4 Fig), common to freshwater lakes and sediment [17, 53, 72], was clearly present with archaea during this time (Fig 6). Furthermore, amplification of *amo*A genes from both AOA and AOB was most pronounced in our winter samples, providing indirect evidence of higher nitrifyer abundance under ice. Although the higher NH_4_^+^ concentrations observed during winter (625 and 540% of top and bottom summer concentrations, respectively) appear to favour high AO, concentrations alone cannot explain the high rates and co-occurrence of AOA and AOB under ice since elevated concentrations were observed at other moments in the year (see Fig 2A). Reduced competition by light-limited phytoplankton for substrate during winter under ice may also help to explain this pattern. Indeed, reduced under-ice chl *a* concentrations compared to summer values is a consistent pattern in Lake Croche (Fig 5) and is similar to observations across ice-covered lakes worldwide [29].

An additional plausible explanation for the co-occurrence of AOB with AOA primarily during winter could be that relief from grazer control enables AOB to reach high abundances under ice, at least in Lake Croche. Within the AO community, AOB may be more vulnerable to predation than AOA given the difference in cell size [8]. It is well known that protistan grazers (e.g., nanoflagellates, ciliates) prefer larger cells [73] and experiments have shown that AOB populations can be controlled directly via grazing, reducing rates of nitrification [74]. A recent review [75] suggests that mortality factors for prokaryotes, such as grazing and viral lysis, may not decline as expected during winter. However, evidence for this claim remains inconclusive and this hypothesis remains to be tested. Bactivorous ciliates and heterotrophic and phototrophic nanoflagellate populations appear to stay relatively abundant during transition periods of ice formation and melt [76, 77], but again grazing measurements were not made under ice or compared across seasons in those studies. Nevertheless, some relief from competition due to ice cover combined with lake mixing that increase substrate availability may create conditions that favour the coexistence of AOA and AOB, making winter under lake ice a control point for nitrification in lakes [25].

High nitrification rates under ice during winter could have critical impacts at the ecosystem scale such as effects on greenhouse gas (GHG) emissions and on spring phytoplankton dynamics given the strong influence that the availability of different N forms has on protistan community structure [78]. Indeed, we observed increased N_2_O saturation under ice in Lake Croche (Fig 5, S2 and S3 Figs) likely resulting in higher emissions during ice-out. In a recent study across boreal lakes, N_2_O accumulation under the ice and evasion after melt could account for approximately 15% of annual emissions from nutrient-poor boreal lakes [26]. Our study confirms that nitrification, at least in systems that remain oxygenated during winter, could be a control point for emissions of this potent GHG. Furthermore, nitrification increases NO_3_^-^ concentrations under ice, resulting in peak concentrations in early spring (Figs 2B, 5A and S3 Fig). Since NO_3_^-^ accumulation under ice is a typical pattern of northern lakes [28], shorter ice duration observed over the last century [30, 31] will likely have impacts on both primary production by reducing the overall NO_3_^-^ availability to prime activity in the spring [79] as well as phytoplankton community structure since NO_3_^-^ is preferentially acquired by diatoms [78, 80]. However, the impact of this effect on phytoplankton community structure as a function of reduced ice-cover remains unexplored.

Our study shows that changes in NH_4_^+^ concentrations influence nitrification rates and shape AO community structure in the surface waters of oligotrophic shield lakes. The pulse of NH_4_^+^ during fall turnover appears to have primed AO activity under the ice where high rates are consistent with reduced competition with phytoplankton, the accumulation of NO_3_^-^ and N_2_O during the ice-covered period, as well as the co-existence of both AOA and AOB. Winter, therefore, appears to be a control point for nitrification in lakes. Despite recent efforts in winter limnology [27–29], this role is poorly understood in the global inland water N cycle and deserves further attention, especially when considering how long-term global warming trends may modify ice duration [81] and the phenology of vertical mixing in lakes [82]. Furthermore, the omnipresence of AOA throughout the year associated with measurable AO rates, also suggests a key role of archaea in the ammonia-oxidizing community of freshwater lakes particularly during periods when NH_4_^+^ concentrations are low.

## Supporting information

S1 Table Informations on ammonia oxidation rates assays. (DOCX)

S2 Table Simple linear regressions between ammonia oxidation rates and environmental variables. (DOCX)

S1 Text Supplementary methods. Additional informations on DNA extraction, PCR amplification, sequencing and phylogenetic analysis.
(DOCX)

S1 Fig Depth interpolated monthly variation in water temperature and dissolved oxygen. (DOCX)

S2 Fig Boxplot of seasonal delta N_2_O variation for the nitrification project. (DOCX)

S3 Fig Variation in NH_4_^+^, NO_3^-^_, N_2_O and chl *a* concentrations from lake sentinel bottom waters. (DOCX)

S4 Fig Phylogenetic relationships. (DOCX)

S1 Data Dataset for the lake sentinel project. (CSV)
