# Supplementary material for: Annual nitrification dynamics in a seasonally ice-covered lake
Source: PLoS One. 2019 Mar 20;14(3):e0213748. doi: 10.1371/journal.pone.0213748 (PMC6426244; doi:10.1371/journal.pone.0213748)
Supplement: S1 Table — (DOCX) [file pone.0213748.s001.docx]

**Supporting Information for**

**Annual nitrification dynamics in a seasonally ice-covered lake**

S1 Table: Incubation times and nutrient concentrations in bottles of ammonia oxidation rates assays. NO_2_^-^, nitrite concentration; NH_4_^+^, *in situ* ammonium concentration; ^15^N-NH_4_^+^, labeled ammonium concentration in bottle; AO, ammonia oxidation; o indicates potential overestimation caused by ^15^N-NH_4_^+^ overshoot > 15%, compared to optimal target (≤ 10% of *in situ* NH_4_^+^)
