# Supplementary material for: Annual nitrification dynamics in a seasonally ice-covered lake
Source: PLoS One. 2019 Mar 20;14(3):e0213748. doi: 10.1371/journal.pone.0213748 (PMC6426244; doi:10.1371/journal.pone.0213748)
Supplement: S2 Table — (DOCX) [file pone.0213748.s002.docx]

**Supporting Information for**

**Annual nitrification dynamics in a seasonally ice-covered lake**

S2 Table. Summary of simple linear regressions using ordinary least squares (OLS) of square root transformed ammonia oxidation rates (nmol N L^-1^ d^-1^) explained by environmental variables. From October 2011 to October 2012 (38 samples). *β*, regression coefficient; R^2^, coefficient of determination; *, *p* < 0.05; **, *p* < 0.001

|  | simple linear regression | |
| --- | --- | --- |
| Variable | *β* | R^2^ |
| NH_4_^+^ (sqrt, µmol L^-1^) | 6.71** | 0.40** |
| NO_3_^-^ + NO_2_^-^ (ln, µmol L^-1^) | 0.97* | 0.15* |
| I_z_ : I_o_ (sqrt) | -11.52 | 0.05 |
| Temperature (ln, °C) | -5.46** | 0.28** |
| Dissolved oxygen (sqrt, mg L^-1^) | 0.34 | 0.02 |
