# Supplementary material for: Annual nitrification dynamics in a seasonally ice-covered lake
Source: PLoS One. 2019 Mar 20;14(3):e0213748. doi: 10.1371/journal.pone.0213748 (PMC6426244; doi:10.1371/journal.pone.0213748)
Supplement: S1 Text — Additional informations on DNA extraction, PCR amplification, sequencing and phylogenetic analysis. (DOCX) [file pone.0213748.s003.docx]

**Supporting Information for**

**Annual nitrification dynamics in a seasonally ice-covered lake**

**S1 Text**

**1. DNA extraction and PCR amplification of *amo*A gene**

DNA was extracted from 0.2 µm pore size Sterivex units using a modified protocol from Zaikova et al. (2010). Briefly, Sterivex units were thawed on ice and then incubated at 37 °C for 1 h in a hybridization oven with 100 µL of lysozyme (125 mg mL^-1^, Sigma^®^ Life Science) and 20 µL of RNaseA (10 µg mL^-1^). Samples were mixed with 100 µL proteinase K (10 mg/mL, Bio Basic Canada inc.) and 100 µL 20% SDS, and incubated a second time at 55°C for 2 h. After proteinase K digestion, the lysate was transferred from the Sterivex unit into centrifuge tubes (2 mL) by introducing air with a 5 mL syringe. We used protein precipitation instead of phenol/chloroform/isoamyl alcohol to eliminate proteins from the lysate by adding 0.583 volume of MPC reagent (Epicentre^®^ Technologies Corporation an Illumina^®^ Company). The mixture was vortexed and centrifuged at 10 000 × g for 10 min at 4°C. The aqueous layer was collected and the DNA precipitated by adding 0.95 volume of isopropanol alcohol. After gentle mixing, the mixture was centrifuged a second time at 10 000 × g for 10 min at 4°C. The DNA pellet was rinsed three times with 750 µL of 70% ethanol, air-dried and resuspended in 25 µL Tris buffer (pH 7.5). To improve amplification efficiency, we added a supplementary step of purification using the PowerClean DNA Clean-Up kit (MO BIO^®^ Laboratories).

Amplification of both archaeal and bacteria *amo*A genes were performed with the following amplification conditions: initial denaturation at 98 °C for 3 min, 35 cycles of 98 °C for 5 sec, annealing at 55 °C for 5 sec, and extension at 72 °C for 10 sec, and final extension at 72 °C for 1 min. PCR were run in a 25 µL reaction mixture that contained 1 µL of purified DNA solution as template, 1.25 µL each 10 nM forward and reverse primers (conc. final 0.5 μM), 0.5 µL of 10 nM dNTP (conc. final 0.20 mM), 0.5 µL Phire Hot Start II DNA Polymerase (Thermo Fisher Scientific Inc.), 5 µl 5X Phire Reaction Buffer (conc. final 1.5 mM MgCl_2_), 1 µL of bovine serum albumin (New England BioLabs^®^ Inc.) for a final concentration of 400 ng µL^-1^, and 13.5 µL of sterile ultra pure water.

**2. Sequencing and phylogenetic analyses**

Clone libraries of archaeal and bacterial *amo*A genes were generated for four and three discrete samplings, respectively, from the Lake Croche time series. Amplicons were visualized on 2 % agarose gels in 1 X TAE, purified using the QIAquick Gel Extraction Kit (Quiagen), and cloned using the CloneJET PCR Cloning Kit with the pJET vector and DH5α chemically competent cells (Thermo Fisher Scientific Inc.). Insert-containing cells were grown overnight at 37 °C on plates containing LB and 100 µg mL^-1^ ampicillin. Ten colonies were selected in each library and examinated for the insert by PCR using Taq polymerase (New England BioLabs^®^ Inc.) and the pJet primers (Thermo Fisher Scientific Inc.). Ten colonies from each sample were screened by determining restriction fragment length polymorphisms (RFLPs). Each 10 µL digestion reaction contained 5 µL of PCR product, 0.13 µL of HhaI (New England BioLabs^®^ Inc.), 1 µL NE buffer 4, and 3.88 µL of sterile ultra pure water. Unique clones were isolated (AOA: 11 clones, AOB: 14 clones) using the GeneJet Plasmid Miniprep Kit (Thermo Fisher Scientific Inc.) and chosen for bidirectional Sanger sequencing (OPERON, Louisville, Kentucky). Nucleotide sequences were trimmed and edited using *MEGA* version 5 (Tamura et al. 2011). The resulting datasets were checked for chimeric sequences with the UCHIME algorithm (Edgar et al. 2011).

**3. References**

Auguet, J.-C., and E. O. Casamayor. 2013. Partitioning of Thaumarchaeota populations along environmental gradients in high mountain lakes. FEMS Microbiol. Ecol. 84: 154-164, doi: 10.1111/1574-6941.12047

Edgar, R. C., B. J. Haas, J. C. Clemente, C. Quince, and R. Knight. 2011. UCHIME improves sensitivity and speed of chimera detection. Bioinformatics 27: 2194-2200, doi: 10.1093/bioinformatics/btr381

Francis, C. A., G. D. O'Mullan, and B. B. Ward. 2003. Diversity of ammonia monooxygenase (amoA) genes across environmental gradients in Chesapeake Bay sediments. Geobiology 1: 129-140, doi:10.1046/j.1472-4669.2003.00010.x

Pester, M., and others. 2012. amoA-based consensus phylogeny of ammonia-oxidizing archaea and deep sequencing of amoA genes from soils of four different geographic regions. Environ. Microbiol. 14: 525-539, doi: 10.1111/j.1462-2920.2011.02666.x

Restrepo-Ortiz, C. X., J.-C. Auguet, and E. O. Casamayor. 2014. Targeting spatiotemporal dynamics of planktonic SAGMGC-1 and segregation of ammonia-oxidizing thaumarchaeota ecotypes by newly designed primers and quantitative polymerase chain reaction. Environ. Microbiol. 16: 689-700, doi: 10.1111/1462-2920.12191

Tamura, K., D. Peterson, N. Peterson, G. Stecher, M. Nei, and S. Kumar. 2011. MEGA5: Molecular evolutionary genetics analysis using maximum likelihood, evolutionary distance, and maximum parsimony methods. Mol. Biol. Evol. 28: 2731-2739, doi: 10.1093/molbev/msr121

Zaikova, E., D. A. Walsh, C. P. Stilwell, W. W. Mohn, P. D. Tortell, and S. J. Hallam. 2010. Microbial community dynamics in a seasonally anoxic fjord: Saanich Inlet, British Columbia. Environ. Microbiol. 12: 172-191, doi: 10.1111/j.1462-2920.2009.02058.x
