# Supplementary material for: Annual nitrification dynamics in a seasonally ice-covered lake
Source: PLoS One. 2019 Mar 20;14(3):e0213748. doi: 10.1371/journal.pone.0213748 (PMC6426244; doi:10.1371/journal.pone.0213748)
Supplement: S1 Fig — (DOCX) [file pone.0213748.s004.docx]

**Supporting Information for**

**Annual nitrification dynamics in a seasonally ice-covered lake**

**S1 Fig**

Depth interpolated monthly variation in A) water temperature and B) dissolved oxygen concentrations (mg/L) between October 2011 and October 2012. White dots are vertical YSI measurements and black dots are sampled depths for nutrients and ammonia oxidation incubations.
