# Supplementary material for: Annual nitrification dynamics in a seasonally ice-covered lake
Source: PLoS One. 2019 Mar 20;14(3):e0213748. doi: 10.1371/journal.pone.0213748 (PMC6426244; doi:10.1371/journal.pone.0213748)
Supplement: S2 Fig — (DOCX) [file pone.0213748.s005.docx]

**Supporting Information for**

**Annual nitrification dynamics in a seasonally ice-covered lake**

**S2 Fig**

Box and whisker plot of delta nitrous oxide (N_2_O) concentrations in water according to the season. Solid horizontal line within each box represents median, box boundaries 25^th^ and 75^th^ percentiles and whisker boundaries 10^th^ and 90^th^ percentiles. Dotted line represents N_2_O concentrations at equilibrium with air.
