# Supplementary material for: Annual nitrification dynamics in a seasonally ice-covered lake
Source: PLoS One. 2019 Mar 20;14(3):e0213748. doi: 10.1371/journal.pone.0213748 (PMC6426244; doi:10.1371/journal.pone.0213748)
Supplement: S3 Fig — (DOCX) [file pone.0213748.s006.docx]

**Supporting Information for**

**Annual nitrification dynamics in a seasonally ice-covered lake**

**S3 Fig**

Temporal variation in bottom water A) ammonium (NH_4_^+^) and nitrate (NO_3_^-^) and B) chlorophyll *a* and delta N_2_O between January 2013 and September 2015 collected as part of lake sentinel project in lake Croche. Dotted line in panel B) represents N_2_O concentrations at equilibrium with air. The period where the lake was covered by ice is depicted by a grey rectangle. Tick mark spacing represent month starting in January.
