# Supplementary material for: Annual nitrification dynamics in a seasonally ice-covered lake
Source: PLoS One. 2019 Mar 20;14(3):e0213748. doi: 10.1371/journal.pone.0213748 (PMC6426244; doi:10.1371/journal.pone.0213748)
Supplement: S4 Fig — (DOCX) [file pone.0213748.s007.docx]

**Supporting Information for**

**Annual nitrification dynamics in a seasonally ice-covered lake**

**S4 Fig**

**
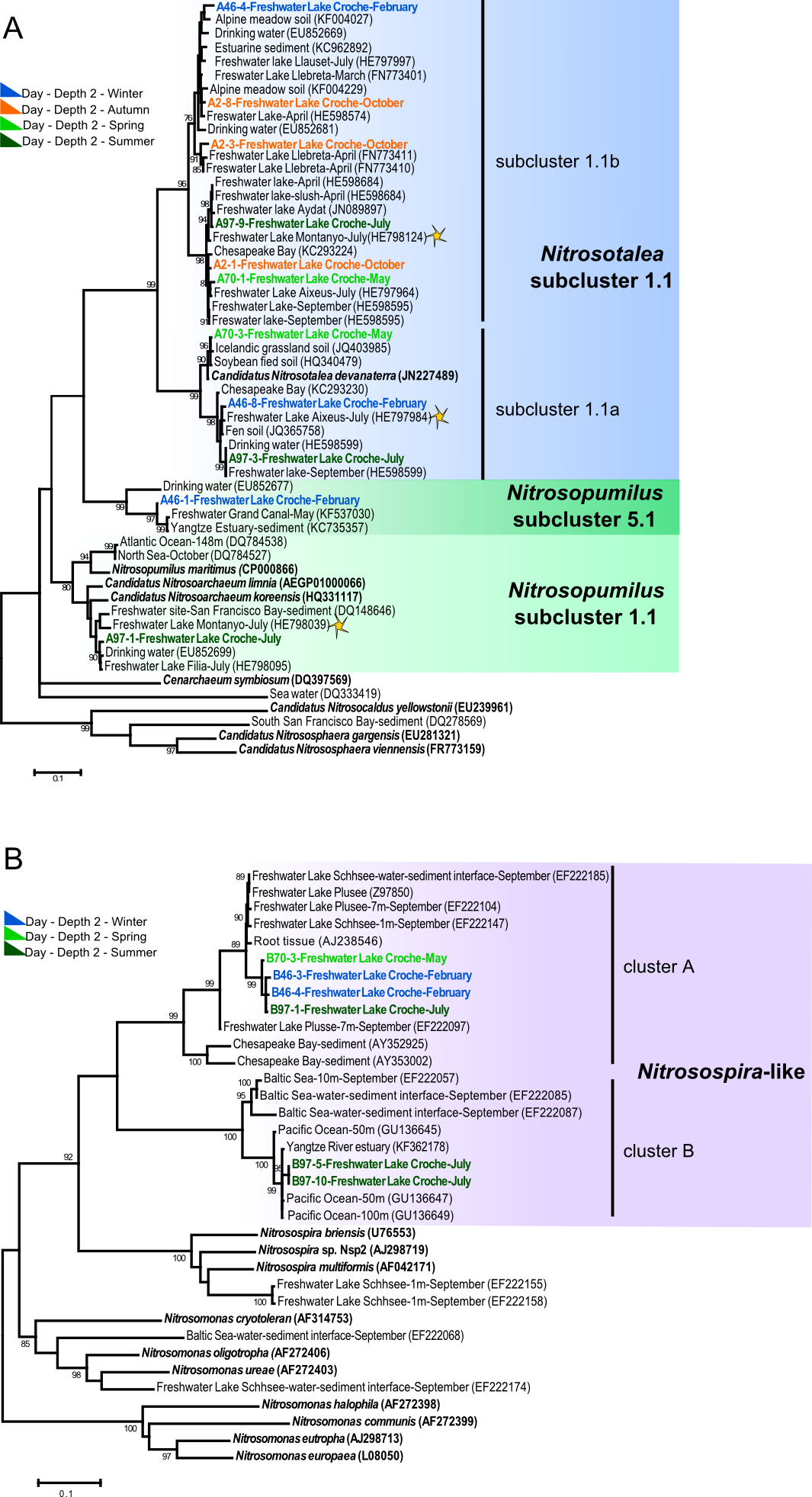
**

Phylogenetic relationships among A) archaeal and B) bacterial *amo*A sequences from Lake Croche (in bold) and previously reported environmental sequences. Nomenclature of archaeal clusters is according to Pester et al. (2012) and Restrepo-Ortiz et al. (2014). Three most abundant AO Thaumarchaeota ecotypes previously found in high mountains lakes (Auguet and Casamayor 2013; Restrepo-Ortiz et al. 2014) are indicated by stars. Nomenclature of bacterial clusters according to Francis et al. (2003). The scale bar represents 0.05 estimated number of substitutions per nucleotide position.
